# Isolation of *Brucella abortus* and *Brucella melitensis* from Seronegative Cows is a Serious Impediment in Brucellosis Control

**DOI:** 10.3390/vetsci5010028

**Published:** 2018-03-09

**Authors:** Mohamed El-Diasty, Gamal Wareth, Falk Melzer, Shawky Mustafa, Lisa D. Sprague, Heinrich Neubauer

**Affiliations:** 1 Animal Health Research Institute-Mansoura Provincial Laboratory, 35516 Mansoura, Egypt; dr_mesbah_m@yahoo.com; 2 Friedrich-Loeffler-Institut, Federal Research Institute for Animal Health, Institute of Bacterial Infections and Zoonoses, 07743 Jena, Germany; falk.melzer@fli.de (F.M.); lisa.sprague@fli.de (L.D.S.); heinrich.neubauer@fli.de (H.N.); 3 Faculty of Veterinary Medicine, Benha University, Moshtohor, 13736 Toukh, Egypt; dr.shawky.gesriha@gmail.com

**Keywords:** *B. abortus*, *B. melitensis*, seronegative cows, isolation, Egypt

## Abstract

Brucellosis is a zoonosis occurring worldwide, with economic and public health impacts. Its diagnosis remains a challenge in endemic countries and basically relies on serology. The present study was carried out on two dairy cattle farms allegedly free from brucellosis, but with sporadic cases of abortion. The aim of this study was to investigate the presence of *Brucella* (B.) spp. in uterine discharge of seronegative cows after abortion. In farm I, *B. melitensis* biovar (bv) 3 was cultured from two of five cows after abortion, while in farm II, *B. abortus* bv 1 was cultured from three of eleven cows after abortion. These cows had been intrauterinely infected but remained seronegative until abortion and seroconverted only thereafter. Shedding of brucellae in uterine discharge of culture positive/seronegative aborting cows is a serious problem resulting in maintenance and further spread of infection. Thus, serosurveys in endemic countries have to be accompanied by molecular detection and/or culture of aborted material to close the diagnostic window and to hinder uncontrolled spread.

## 1. Introduction

Brucellosis is a highly contagious bacterial disease of zoonotic importance causing significant reproductive loss due to high rates of abortion and infertility. In Egypt, the disease is endemic nationwide in livestock and humans. The disease has been reported in cattle, buffaloes, sheep, goats, and camels, and *Brucella* (B.) has been isolated from Nile cat fish and carrier hosts such as dogs and cats [1,2]. Diagnosis of animal brucellosis is still challenging in Egypt and relies on serology using direct agglutination tests. Despite the implementation of a control program, i.e., test-and-slaughter of seropositive animals and vaccination of free herds, the disease is re-emerging in different regions. Intrauterinely infected calves seroconvert only after giving birth or after abortion and then shed brucellae in high numbers [3]. Despite the effort and money spent on surveillance programs against brucellosis, these seronegative animals hamper the control program and might facilitate spillover in to the environment and spread the disease to susceptible hosts. Thus, the aim of the current work was to investigate the presence of *Brucella* in uterine discharge and fetal fluid from seronegative cows that had aborted.

## 2. Finding and Discussion

The current study was conducted in two dairy cattle farms allegedly free from brucellosis according to the routine periodic examination applied by General Organization of Veterinary Services (GOVS) in two different governorates. Farm I is located in Damietta, and had a history of brucellosis three years ago, while farm II is located in Ismailia, and was without any known history of brucellosis. The farm owners agreed to participate. The two farms are involved in the national brucellosis control program and had not applied any vaccination program before. They were assumed to be free of brucellosis based on routine serological examination carried out by GOVS. The program includes examination of all animals with Rose-Bengal Plate Test (RBPT), Buffered Acidified Plate Antigen Test (BAPAT) and use of the complement fixation test (CFT) and Enzyme-linked Immunosorbent Assay (ELISA) as confirmatory test. In the last quarter of 2015, both farms tested seronegative using RBPT, BAPAT and CFT. *Brucella* antigens used in the study were obtained from the Veterinary Serum and Vaccine Research Institute, Abbasia, Cairo, Egypt. In December 2015, five cows were aborted in farm I and eleven in farm II, respectively. Uterine discharge from all aborting and contact cows that gave birth without complication was collected at each farm and subjected to *Brucella* isolation and identification. There was no chance to collect samples from aborted fetuses due to their disposal before sampling. Identification and biotyping of *Brucella* isolates was carried out using classical biotyping methods [4]. Briefly, swabs were plated directly on three different agars (blood agar, *Brucella* agar media and *Brucella* selective agar media). A single colony was selected from the *Brucella* selective agar media and sub-cultured again on *Brucella* selective agar media to obtain uncontaminated colonies. After overnight incubation, one colony was picked and submitted to Matrix Assisted Laser Desorption/Ionization (MALDI-TOF) for genus *Brucella* identification, as described previously [5]. Genomic DNA was extracted from heat inactivated biomasses of all confirmed *Brucella* colonies using the High Pure template preparation kit (Roche Applied Sciences, Mannheim, Germany) according to the manufacturer’s instructions. Molecular identification of isolates at species level as *B. abortus* and *B. melitensis* was confirmed by AMOS-PCR, as described previously [6].

In farm I, *B. melitensis* bv 3 was isolated from the uterine discharge of two cows of the five who had aborted. In farm II, *B. abortus* bv 1 was recovered from uterine discharges of three cows of the eleven who had aborted. *B. melitensis* bv 3 and *B. abortus* bv 1 are the predominant *Brucella* biotypes in Egypt [2]. Accurate diagnosis of brucellosis requires either isolation of bacteria or molecular detection of DNA and/or strong positive serological reactions accompanied by prominent clinical signs, e.g., fever and abortion. The two farms included in this study showed cases of abortion after routine testing with RBPT, BAPAT and CFT with negative results. Identification of seronegative carriers can be achieved by analyzing vaginal exudates or milk [7]. Detection of *Brucella* spp. DNA in semen of seronegative bulls has previously been reported [8], and in milk of seronegative cows [9,10] has previously been reported. *Brucella* spp. has been recovered from milk came from seronegative cows [11] and from blood, bone marrow, lymph nodes and vaginal exudates of seronegative cattle and goats [12,13,14]. Recently, *B. abortus* bv1 was recovered from vaccinated dairy cattle herd allegedly free from brucellosis in Egypt [15]. Culture-positive/seronegative cases can be expected during seroconversion. The presence of a small number of bacteria in the blood stream is not able to stimulate a humoral immunological response, and is unable to generate antibodies (or induce abortion) and will result in the negative response to serological diagnosis. These facts can explain the absences of antibody titers in infected cases. It is worth mentioning that serological examination of animals directly after abortion is of no value, as the antibodies against *Brucella* are detectable at the earliest 14 days after the onset of infections.

In cases with individual low antibody concentrations or no antibodies, animals often present with false negative results [16], but are very likely to be infectious [7]. In these cases, DNA and bacteria circulate in the blood and are present in tissue and may therefore be detected with molecular diagnostics or via isolation. The keeping of false negative animals is harmful for healthy livestock and the environment, and impedes surveillance and control programs. Thus, diagnosis of brucellosis should be always complemented by bacteriological and molecular diagnosis in herds at risk [17]. Although seronegative/culture positive cases have been reported several times, worldwide, it is nevertheless underestimated in Egypt, resulting in serious impediment to brucellosis control. This finding highlights the importance of isolation and molecular detection of the organism in countries where brucellosis is endemic, despite the high sensitivity and specificity of serological tools.

## 3. Conclusions

While false positive animals only affect the economy of the herd, due to the excessive disposal of animals, the presence of infected seronegative cattle is much more problematic for the economy and healthy development of herds, and is harmful for human health and environment. Considering the findings of this study and the literature presented, we recommend the use of different serological assays together with PCR and/or isolation of bacteria from all animals following abortion and contact animals as a routine for the detection of *Brucella* spp. in endemic countries like Egypt. Serosurveys of brucellosis in endemic countries have to be accompanied by molecular detection and isolation.
